# Supramolecular Fluorescence Probe Based on Twisted Cucurbit[14]uril for Sensing Fungicide Flusilazole

**DOI:** 10.3389/fchem.2019.00154

**Published:** 2019-03-21

**Authors:** Ying Fan, Rui-Han Gao, Ying Huang, Bing Bian, Zhu Tao, Xin Xiao

**Affiliations:** ^1^State Key Laboratory Breeding Base of Green Pesticide and Agricultural Bioengineering, Key Laboratory of Green Pesticide and Agricultural Bioengineering, Ministry of Education, Guizhou University, Guiyang, China; ^2^Key Laboratory of Macrocyclic and Supramolecular Chemistry of Guizhou Province, Guizhou University, Guiyang, China; ^3^College of Chemical and Environmental Engineering, Shandong University of Science and Technology, Qingdao, China

**Keywords:** twisted cucurbit[14]uril, thioflavin T, fluorescent probe, triazole fungicides, flusilazole

## Abstract

The host-guest complex of the common dye, thioflavin T (ThT), and twisted cucurbit[14]uril (*t*Q[14]) was selected as a fluorescent probe to determine non-fluorescent triazole fungicides, including flusilazole, azaconazole, triadimefon, tebuconazole, tricyclazole, flutriafol, penconazole, and triadimenol isomer A, in an aqueous solution. The experimental results reveal that the ThT@*t*Q[14] probe selectively responded to flusilazole with significant fluorescence quenching and a detection limit of 1.27 × 10^−8^ mol/L. In addition, the response mechanism involves not only a cooperation interaction—ThT occupies a side-cavity of the *t*Q[14] host and the triazole fungicide occupies another side-cavity of the *t*Q[14] host—but also a competition interaction in which both ThT and the triazole fungicide occupy the side-cavities of the *t*Q[14] host.

## Introduction

**Figure 1:**
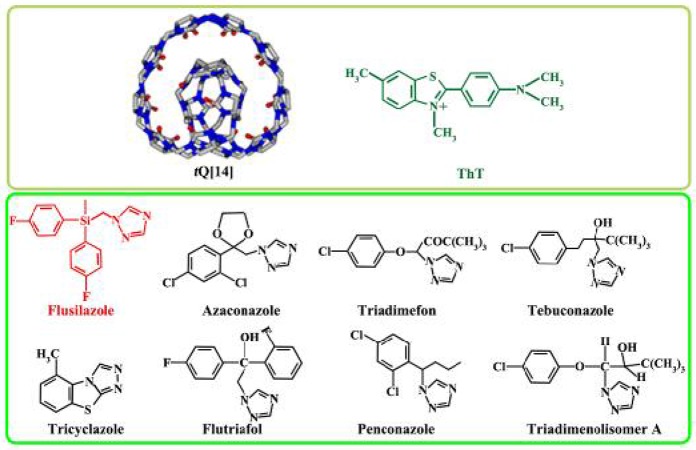
Structures of *t*Q[14], ThT, and eight fungicide triazoles, namely flusilazole, azaconazole, triadimefon, tebuconazole, tricyclazole, flutriafol, penconazole, and triadimenol isomer A.

Triazole systemic fungicides, particularly flusilazole (1-{[*bis*(4-fluorophenyl) (methyl)silyl]methyl}-1*H*-1,2,4-triazole), are widely used in fruit, vegetable, and grain crops during cultivation and storage with high efficiency and good sterilization ability (Figure 1) (Zhang Y. H. et al., 2016). However, they still have somewhat toxic or other undesirable side effects on non-target organisms. Extensive or inappropriate use can cause soil and water pollution, and thus threaten human health (Ma et al., 2016). Therefore, it is necessary to develop sensitive and selective methods for the analysis of triazole fungicides, which are usually present in trace amounts.

The most common analytical method used for the trace determination of triazoles, particularly flusilazole residues in water, is chromatography e.g., liquid chromatography-tandem mass spectrometry (LC-MS/MS) (García-Valcárcel and Tadeo, 2011; Fu et al., 2017), high-performance liquid chromatography (HPLC) with ultraviolet light, diode-array detection (DAD), photodiode-array (PDA) detection (Bordagaray et al., 2013, 2014; Qi et al., 2014; Ma et al., 2016; Zhang Y. H. et al., 2016), gas chromatography (GC) with nitrogen-phosphorous detection (NPD) or electron capture detection (ECD) (Lozowicka et al., 2015; Im et al., 2016), and GC-mass spectrometry (GC-MS) (Tseng et al., 2014; Chu et al., 2015), and with tandem MS (GC-MS/MS) (Xu et al., 2013). Nevertheless, these chromatographic techniques necessitate experienced workers, costly devices, and lengthy specimen preparation. In comparison with the above-mentioned methods, spectrofluorimetry is the most favorable analytical strategy employed to analyze different biological specimens due to its innate simplicity, high sensitivity, and accessibility in the majority of quality-control and clinical laboratories (Yao et al., 2013). However, determination of the existence of many common triazoles cannot be performed directly using typical fluorimetric techniques since the aqueous solutions with triazoles do not have native fluorescence.

It is noteworthy that the complexation of a fluorescent dye with a macrocycle host can induce significant change in its fluorescence (Zhou et al., 2008; Zhang et al., 2012; Kogawa et al., 2014). Such assays depend on an indicator-displacement technique in which the analyte competitively supplants a fluorescent guest that results in an alteration in its fluorescence intensity (You et al., 2015; Sayed and Pal, 2016). Various macrocyclic compounds, including cyclodextrins, calix[*n*]arenes, and pillar[*n*]arenas, have long been employed as the hosts in several macrocyclic-dye supramolecular systems (Choudhury et al., 2010; Lau and Heyne, 2010; Liu et al., 2015b; Sun et al., 2015). In particular, cucurbit[*n*]urils (Q[*n*]s) are made up of n glycoluril units connected via 2n methylene bridges (Day et al., 2000; Kim et al., 2000; Cong et al., 2016). These hosts have highly polar carbonyl-fringed portals with hydrophobic cavities that can create remarkably stable complexes with different guest molecules (Kim, 2002; Dsouza et al., 2011; Gao et al., 2017; Liu J. et al., 2017; Murray et al., 2017; Yang et al., 2018). The origination of inclusion complexes frequently improves or interrupts the photo-physical and photo-chemical characteristics of the guest molecules. For example, using different Q[*n*]s to encapsulate some dyes can change the fluorescent characteristics of the guest molecules (Praetorius et al., 2008; Baumes et al., 2009, 2011; Choudhury et al., 2009). The dye@Q[*n*] complexes can be composed of supramolecular fluorescent probes with high sensitivity and selectivity to recognize and detect analytes, such as metal ions, amines, pesticides, drugs, and DNA (Wheate, 2008; Mohanty et al., 2009; Nau et al., 2009; Zhou et al., 2009; Day and Collins, 2012; Xing et al., 2013; Elbashir and Aboul-Enein, 2015; Liu W. Q. et al., 2017; Xi et al., 2017; Tang et al., 2018; Wang et al., 2018).

Thioflavin T, 3,6-dimethyl-2-(4-dimethylaminophenyl) benzthiazolium cation (ThT) is a benzthiazolium dye (Figure 1). An aqueous solution of ThT reveals a weak native fluorescence. Nevertheless, as revealed by previous evaluations, the fluorescence of ThT in an aqueous solution can be vastly improved in the presence of Q[7], Q[8], and twisted cucurbit[14]uril (*t*Q[14]). It was found that ThT can form ThT@Q[*n*] inclusion complexes with different stoichiometric ratios, which can be applied to identify both alkali-metal and alkaline-earth-metal ions (Choudhury et al., 2009, 2010; Mohanty et al., 2009; Wang et al., 2018). In the present study, a procedure was proposed to determine the residues of triazole fungicides, such as azaconazole, triadimefon, tebuconazole, tricyclazole, flutriafol, penconazole, and triadimenol isomer A in aqueous solution (Figure 1).

## Results and Discussion

### Fluorescence Quenching of ThT@*t*Q[14] With Flusilazole

**Figure 2:**
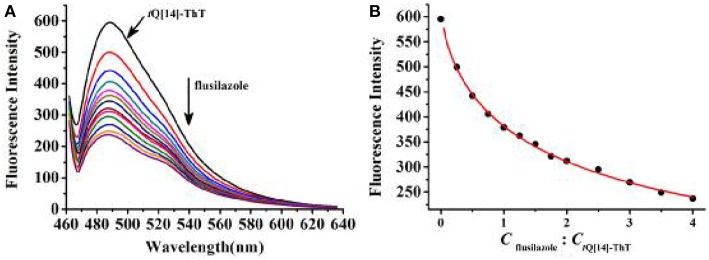
**(A)** Fluorescence titration spectra (λ_ex_ = 448 nm) for ThT@*t*Q[14] (1:1, 1 μM) in the presence of flusilazole of the following different stoichiometries in μM: 0, 0.25, 0.5, 0.75, 1.0, 1.25, 1.5, 1.75, 2.0, 2.5, 3.0, 3.5, and 4.0; **(B)** Plot obtained for fluorescence intensity of ThT@*t*Q[14] vs. C_flusilazole_/C_ThT@t*Q*[14]_.

**Figure 3:**
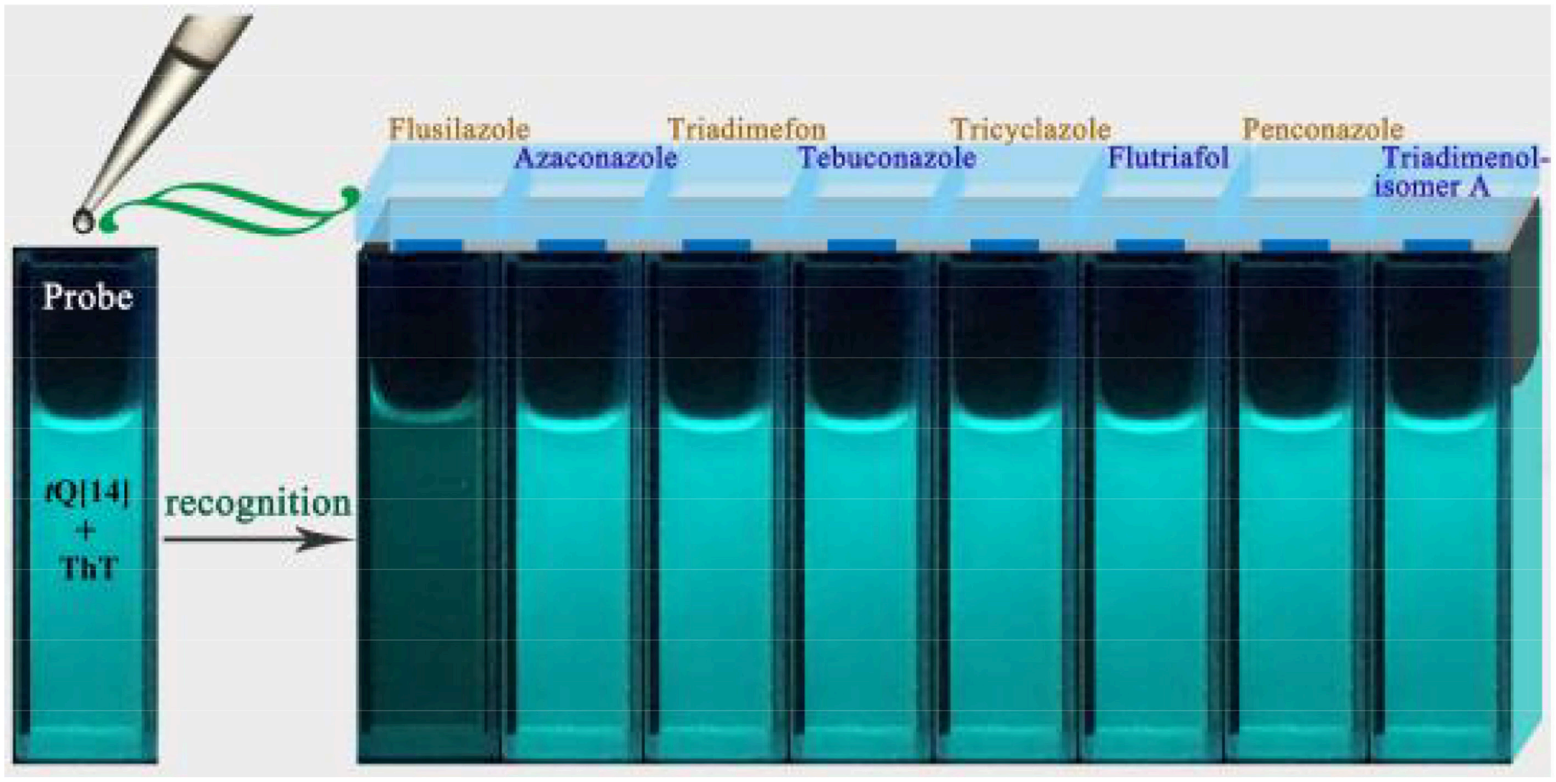
Fluorescence images of ThT@*t*Q[14] (50 μM) upon addition of eight triazole pesticides (50 μM) under UV-lamp irradiation (365 nm).

Previous studies have proven that the fluorescence of ThT in an aqueous solution over a wide range of interaction ratios can be enhanced by its interaction with *t*Q[14] via different interaction models due to the multiple cavity features of the *t*Q[14] molecule and two active moieties (the benzothiazole and dimethylaminobenzene moieties) in ThT (Wang et al., 2018). In the current work, we focused on the inclusion complexes of ThT@*t*Q[14] with 1:1 and 1:5 interaction ratios in the presence of the eight selected triazoles, the corresponding fluorescence spectra of which can be seen in Figure 2 and Figures S1–S3. A significant fluorescence quenching of the ThT@*t*Q[14] inclusion complex probe (1:1) was only observed upon increasing the amount of flusilazole; ~60% of the fluorescence intensity was lost when C_flusilazole_/C_ThT@t*Q*[14]_ was ~4 (Figure 2A). Figure 2B shows the plot of the fluorescence intensity at λ_em_ = 488 nm upon increasing the flusilazole concentration; K_observed_ was calculated by the nonlinear-least-squares method to be (8.2 ± 0.4) × 10^5^ L/mol obtained (Figure S4), which is likely due to the influence of the interaction of *t*Q[14] with flusilazole on the interaction of *t*Q[14] with ThT. Moreover, the ThT@*t*Q[14] fluorescent probe presented high selectivity for flusilazole because the probe showed little or almost no response to the other seven triazole fungicides studied (Figure 3 and Figure S1). The ThT@*t*Q[14] inclusion complex probe at a molar ratio of 1:5 was also selected to investigate its response to triazole pesticides with the addition of flusilazole also leading to a fluorescence quenching of the ThT@*t*Q[14] inclusion complex probe; only ~22% of the fluorescence intensity was lost when C_flusilazole_/C_ThT@t*Q*[14]_ was ~4 (Figure S2). However, the ThT@*t*Q[14] fluorescent probe also showed little or almost no response to the other triazole fungicides studied (Figure S3).

### Analytical Characteristics of ThT@*t*Q[14] Probe

The standard calibration curve (Figure S5) of the complexes of flusilazole with ThT@*t*Q[14] (1:1) was obtained using the plot of fluorescence intensity *F* compared to flusilazole concentration. The linear range was (0.0–1.0) × 10^−6^ mol/L for flusilazole with a correlation coefficient of 0.9888. For flusilazole, the limit of detection (LOD) was 1.27 × 10^−8^ mol/L and the linear regression equation *F* = −216.24*C*+576.26. Although the ThT@*t*Q[14] (1:5) inclusion complex was less sensitive to flusilazole, it still exhibited a very low LOD (2.80 × 10^−8^ mol/L) and the linear regression equation was *F* = −98.25*C*+863.26 (Figure S6).

### Effects of Interfering Substances

In the present work, the impacts of different common interfering substances on the determination of flusilazole using the ThT@*t*Q[14] probe were also investigated. The tolerance limit was established as the concentration of an added interfering substance that causes a relative error of < ±5% in the flusilazole determination. The samples consisted of a fixed amount of flusilazole (1.0 μM) and ThT@*t*Q[14] (1.0 μM) with an increasing amount of different interfering substances; the corresponding results are shown in Table 1 and Figure S7. It is clear that the determination was free from interference in the presence of the common metal ions and anions in aqueous solutions, except Ca^2+^. Structural analysis revealed that *t*Q[14] had a similar portal size to those of Q[6] and Q[7] (Cheng et al., 2013; Liu et al., 2015a; Li et al., 2016; Zhang J. et al., 2016; Zhang et al., 2017), and a higher portal carbonyl intensity (Cheng et al., 2013; Liu et al., 2015a; Zhang J. et al., 2016; Zhang et al., 2017), which could exhibit a higher affinity for metal cations, particularly the Ca^2+^ cation (Cheng et al., 2013; Qiu et al., 2017; Wang et al., 2018). The titration fluorescence spectra recorded for the *t*Q[14]-ThT-flusilazole (1:1:1) system upon increasing the amount of Ca^2+^ showed that the fluorescence of the ternary interaction system could be further decreased due to the influence of the Ca^2+^ cation (Figure S8). The titration ^1^H nuclear magnetic resonance (NMR) spectra for the *t*Q[14]-ThT-flusilazole (1:1:1) system in the presence of Ca^2+^ also showed that the guest molecules, ThT and flusilazole, were gradually pushed out from the cavity area upon increasing the amount of Ca^2+^ (Figure S9).

**Table 1:** Impact of interfering substances (tolerance error ± 5%).

**Interfering**	**Tolerance (mol/L)**	**Relative error (%)**
Na^+^	6.0 × 10^−5^	−3.38
K^+^	5.0 × 10^−5^	−1.94
Zn^2+^	1.0 × 10^−3^	−2.65
Mg^2+^	1.0 × 10^−3^	−2.45
Cu^2+^	5.0 × 10^−5^	−4.67
Cl^−^	4.0 × 10^−5^	−3.97
Br^−^	5.0 × 10^−5^	−1.38
${\text{NO}}_{3}^{-}$	6.0 × 10^−5^	−4.63
${\text{HSO}}_{4}^{-}$	5.0 × 10^−5^	−1.38
H_2_${\text{PO}}_{4}^{-}$	4.0 × 10^−5^	−2.77

### Preliminary Exploration of the Response Mechanism of ThT@*t*Q[14] Fluorescent Probe Toward Flusilazole

#### Titration ^1^H NMR Spectra

**Figure 4:**
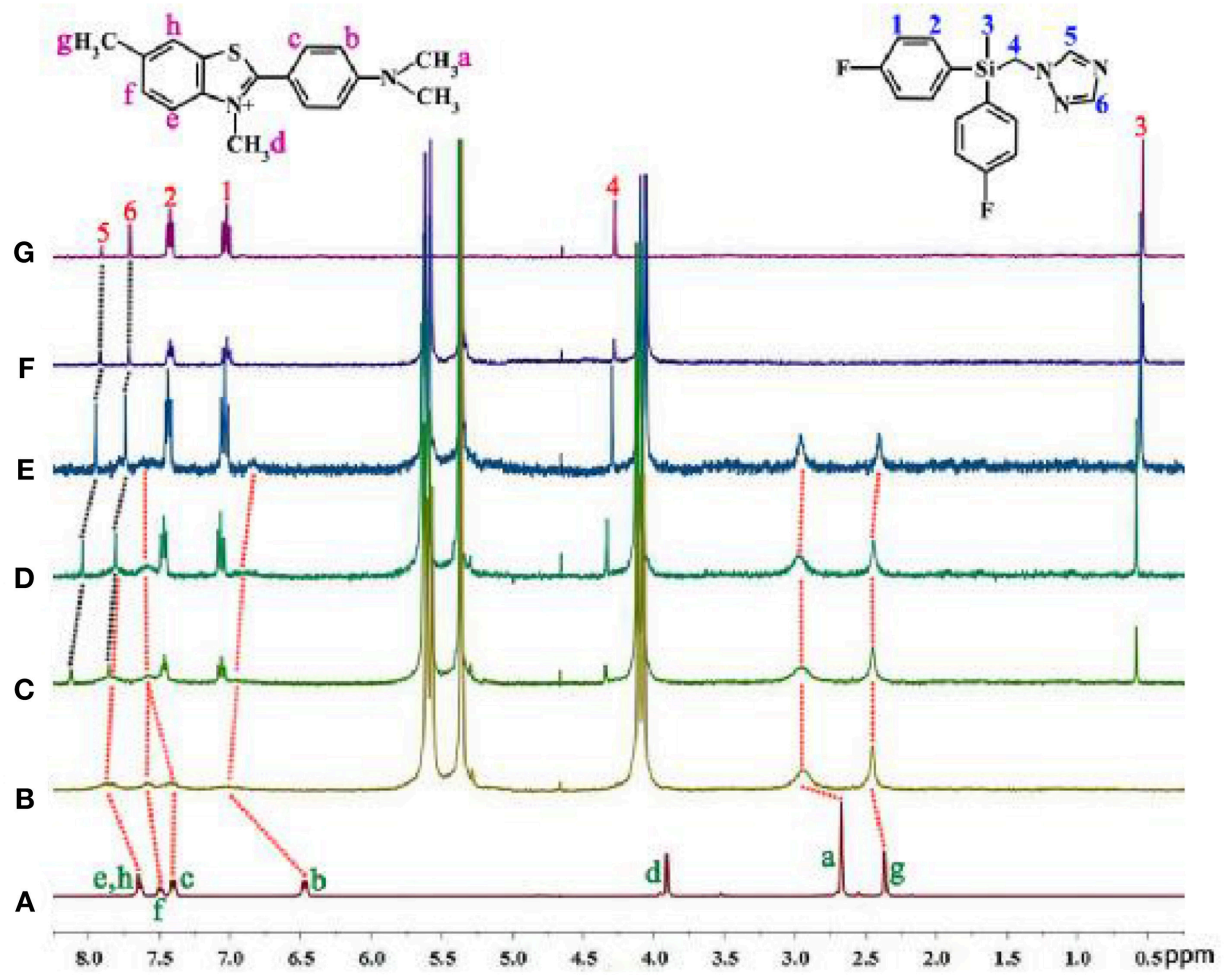
^1^H NMR spectra (400 MHz, D_2_O) for **(A)** ThT, **(B)** *t*Q[14]-ThT (1:1), **(C)** *t*Q[14]-ThT-flusilazole (1:1:0.5), **(D)** *t*Q[14]-ThT-flusilazole (1:1:1), **(E)** *t*Q[14]-ThT-flusilazole (1:1:2), **(F)** *t*Q[14]-flusilazole, and **(G)** flusilazole.

We have found that the ThT@*t*Q[14] fluorescent probe was selectively sensitive toward flusilazole and could be used to detect flusilazole via a significant fluorescence quenching process. Why is the ThT@*t*Q[14] fluorescent probe selectively sensitive to flusilazole? How does the flusilazole molecule influence the interaction between the ThT molecule with the *t*Q[14] host molecule? What could the interaction mode be? To understand the selectivity and response mechanism of the ThT@*t*Q[14] fluorescent probe toward flusilazole, titration ^1^H NMR spectra were recorded upon the gradual addition of one of the selected triazole fungicides into the solution of the ThT@*t*Q[14] (1:1) fluorescent probe. The detailed interaction of ThT and *t*Q[14] has been discussed in a previous work (Figures 4A,B) (Wang et al., 2018). Figure 4F shows the ^1^H NMR spectrum for the flusilazole in the presence of *t*Q[14]. When compared with the ^1^H NMR spectrum for the unbound flusilazole molecule (Figure 4G), the proton resonances corresponding to H5 and H6 on the triazole moiety of the bound flusilazole molecule underwent slightly down-field shifts (0.01 and 0.01 ppm, respectively), whereas the other proton resonances experienced slightly up-field shifts, suggesting that a weak interaction existed between *t*Q[14] and the flusilazole guest molecule or π··· π stacking of the aryl groups of the flusilazole guest molecules. Figures 4C–E show the titration ^1^H NMR spectra for the *t*Q[14]-ThT complex upon increasing the amount of flusilazole. The addition of flusilazole appeared to pull the bound ThT out of the interaction area of the *t*Q[14] host because the proton resonances were closer to those of the unbound ThT molecule. However, the proton resonances of flusilazole, in particular, the H5 and H6 experienced down-field shift, suggesting that ThT was still interacting with the host and flusilazole was located in the de-shielding region of *t*Q[14] (probably at portal area). Similar phenomena could be observed for the other *t*Q[14]-ThT-triazole fungicide systems, in which the addition of the triazole fungicide could lead to a change in the interaction between *t*Q[14] and ThT. Moreover, both ThT and the triazole fungicide exhibited an interaction with the *t*Q[14] host (Figures S10–S16). At first, it seemed that it was difficult to obtain the required information using the titration ^1^H NMR method. A closer inspection revealed that the addition of different triazole fungicides could lead to different chemical shifts for the proton resonances corresponding to the ThT molecule, and the effects of the different triazole fungicides on the bound ThT were compared with the chemical shift corresponding to proton Hg. The data in Table 2 reveal that flusilazole resulted in the larger change in the chemical-shift values (Δδ = 0.05) when the *t*Q[14]-ThT-triazole fungicide ratio was 1:1:2.

**Table 2:** Chemical shifts corresponding to proton Hg of ThT.

**Guest**	**δ [Q14-ThT (1:1)]**	**δ [Q14-ThT-G (1:1:2)]**	**Δδ**
Flusilazole	2.469	2.419	0.050
Azaconazole	2.453	2.439	0.014
Triadimefon	2.458	2.429	0.029
Tebuconazole	2.449	2.422	0.027
Tricyclazole	2.450	2.432	0.018
Flutriafol	2.453	2.429	0.024
Penconazole	2.451	2.430	0.021
Triadimenol isomer A	2.457	2.437	0.020

2D diffusion-ordered NMR spectroscopy (DOSY) experiments were performed to afford further evidence for the formation of the *t*Q[14]-ThT-flusilazole (1:1:1) ternary interaction species. Figure S17 depicts the DOSY spectra of *t*Q[14], ThT, flusilazole, and *t*Q[14]-ThT-flusilazole (1:1:1) ternary interaction species in D_2_O at 298 K and the corresponding diffusion coefficients (D) are 3.49 × 10^−10^, 5.46 × 10^−10^, 3.88 × 10^−10^, and 2.92 × 10^−10^, respectively. According to the values of four species, the *t*Q[14]-ThT-flusilazole (1:1:1) ternary interaction species is the smallest, suggesting that the ternary species could be the largest species, moreover, all the proton signals of the host and the guest display the same diffusion coefficient (D = 2.92 × 10^−10^ · m^2^ · s^−1^), indicating that they are part of the same species.

#### Isothermal Titration Calorimetry

Isothermal titration calorimetry (ITC) experiments were conducted to determine the association constants and thermodynamic parameters of the host-guest interaction between *t*Q[14] and ThT with the triazole fungicides in an aqueous solution to further explore the fluorescence-quenching mechanism of ThT@*t*Q[14] with flusilazole (Figures S18–S26). From the data acquired (Table 3), all the association constants obtained for the triazole fungicides@*t*Q[14] complexes [(1.46–8.39) × 10^5^ L/mol] were slight larger than that of the ThT@*t*Q[14] complex (1.28 × 10^5^ L/mol), but there are no significant differences. The titration ^1^H NMR study has proved that these pesticides could not replace ThT to form pesticide@*t*Q[14] inclusion complexes.

**Table 3:** Thermodynamic parameters of *t*Q[14] and ThT with eight triazole pesticides in aqueous solution at 298.15 K.

**Complex**	***Ka* (m^**−1**^)**	**Δ*H*(kJ/mol)**	***T*Δ*S*(kJ/mol)**
*t*Q[14]-ThT	(1.28 ± 0.03) × 10^5^	−48.4	−19.2
*t*Q[14]-flusilazole	(8.39 ± 0.05) × 10^5^	−38.4	−4.6
*t*Q[14]-azaconazole	(5.45 ± 0.08) × 10^5^	−36.8	−4.0
*t*Q[14]-triadimefon	(1.46 ± 0.04) × 10^5^	−64.3	−34.9
*t*Q[14]-tebuconazole	(3.00 ± 0.09) × 10^5^	−42.7	−11.4
*t*Q[14]-tricyclazole	(5.01 ± 0.12) × 10^5^	−36.8	−4.2
*t*Q[14]-flutriafol	(1.97 ± 0.05) × 10^5^	−64.6	−34.3
*t*Q[14]-penconazole	(5.23 ± 0.08) × 10^5^	−38.8	−6.2
*t*Q[14]-triadimenol isomer A	(4.31 ± 0.10) × 10^5^	−39.0	−6.9

**Figure 5:**
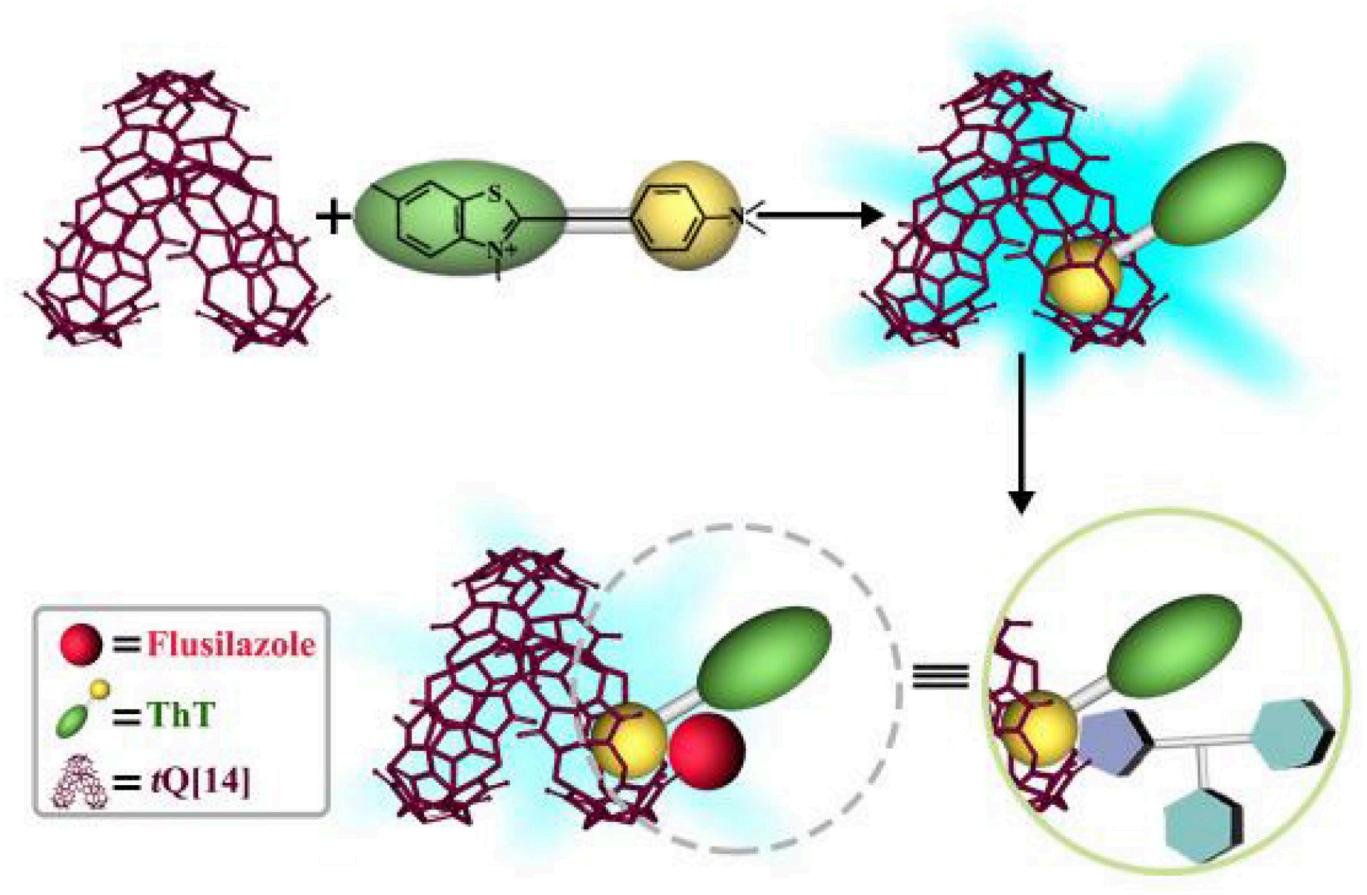
Possible response mechanism for fluorescent probe ThT@*t*Q[14] with flusilazole.

The fluorescence enhancement mechanism of ThT could be due to the restriction of the freely rotating dimethylamine group on the benzene moiety, making the lone pair electrons on nitrogen atoms conjugate to the ThT aromatic system. The shell-like cavity structure of *t*Q[14] can provide such controlled environment, the inclusion of dimethylamino phenyl moiety of ThT inhibited dimethylamino free rotation, moreover, the shell-like cavity structure of *t*Q[14] could prevent ThT from threading through the side cavity of *t*Q[14] (Liu et al., 2015a; Li et al., 2016). Therefore, we can observe ThT fluorescence enhancement. Unlike HMeQ[6] and Q[7] which have similar portal sizes to that of the side portals of *t*Q[14], ThT could thread through their cavities, and show a weak fluorescence emission (referring the fluorescence spectra as shown in Figure S27). On the other hand, the fluorescence emission of ThT is also affected by its electron-pushing group (dimethylamine group) and electron-withdrawing group (quaternary ammonium moiety). The titration ^1^H NMR spectra could provide the interaction images of this ternary system: the flusilazole was located in the de-shielding region of *t*Q[14] (probably at portal area), which caused the chemical shift change of ThT proton resonance to move toward the free ThT, in particular, the interaction of the azole moiety and quaternary ammonium moiety could weaken the electron-withdrawing capacity of quaternary ammonium moiety, resulting in partial quenching of the fluorescence of the *t*Q[14]-ThT-flusilazole interaction system (Figure 5). According to the suggested interaction mode, whether the fluorescence quenching of ThT@*t*Q[14] is caused mainly depends on the interaction of the azole moiety of pesticides and quaternary ammonium moiety of ThT and the ability of pesticides to pull ThT out of the side cavity of *t*Q[14]. While the NMR and ITC measurement results showed that the flusilazole has the biggest impact, although the differences from other triazole fungicides are subtle.

## Conclusions

In order to further expand the application of Q[*n*]-based host-guest chemistry, especially the host-guest complexes with fluorescent properties, in detection and recognition, a known host-guest complex of ThT@*t*Q[14] (1:1) was used as a fluorescent probe to determine non-fluorescence triazole fungicides, including flusilazole, azaconazole, triadimefon, tebuconazole, tricyclazole, flutriafol, penconazole, and triadimenol isomer A. This new and simple fluorometry method proved to be highly selective and sensitive to one of triazole fungicides—flusilazole, and the determination was free from interference by the common metal ions and anions in aqueous solutions, except Ca^2+^. The investigation of the response mechanism revealed that a side-cavity of the *t*Q[14] host includes the dimethylamino phenyl moiety of ThT, resulting in the ThT fluorescence enhancement; the addition of flusilazole results in the interaction of the azole moiety of flusilazole and quaternary ammonium moiety of ThT, which could weaken the electron-withdrawing capacity of quaternary ammonium moiety, resulting in partial quenching of the fluorescence of the *t*Q[14]-ThT-flusilazole interaction system. This unusual phenomenon results from the novel structural feature of *t*Q[14], namely that *t*Q[14] possesses a central-cavity and two of the same side-cavities.

## Experiment

### Materials

*t*Q[14] was set up and purified in our laboratory according to a procedure detailed in the literature (Cheng et al., 2013). Analytical grade flusilazole (99.5%), azaconazole (99.5%), triadimefon (99.0%), tebuconazole (99.0%), tricyclazole (99.0%), flutriafol (98.6%), penconazole (99.5%), and triadimenol isomer A (95.4%) were purchased from Dr. Ehrenstorfer GmbH (Augsburg, Germany) and used as-received without any further purification. Double-distilled water was used for each of the experiments.

### ^1^H NMR

Each of the ^1^H NMR spectra, including those for titration experiments, were documented at 25°C on a JEOL JNM-ECZ400s spectrometer using SiMe_4_ as an internal reference. D_2_O was utilized as a field-frequency lock and the chemical shifts documented in parts per million (ppm).

### ITC

Microcalorimetric experiments were conducted with a Nano ITC (TA, USA) isothermal titration calorimeter. Then, 25 consecutive 10-μL aliquots of a 1 mM *t*Q[14] solution were introduced into the microcalorimetric reaction cell, which contained 1.3 mL of a 0.1-mM guest molecule solution at 25°C. The heat of reaction was corrected for the heat of dilution of the guest molecule solution, which was determined in a separate experiment. Each of the solutions were de-gassed before the titration via ultrasonication. Computer simulations (curve fitting) were conducted with Nano ITC analysis software.

### Fluorescence Titration

The fluorescence spectra of the host-guest complexes were documented at 25°C using a Varian Cary Eclipse spectrofluorometer (Varian, Inc., Palo Alto, CA, USA). Stock solutions of *t*Q[14] (1 × 10^−3^ mol L^−1^), ThT (1 × 10^−3^ mol L^−1^), and flusilazole (1 × 10^−4^ mol L^−1^) were set up in water. Working solutions were set up by diluting the stock solutions to the necessary concentrations.

A *t*Q[14]-ThT (1:1) complex solution was set up at a fixed concentration of 1 × 10^−6^ mol·L^−1^ in H_2_O, which was subsequently combined with flusilazole at guest/host ratios of 0, 0.25:1, 0.5:1, 0.75:1, 1:1, …, and 4:1. Fluorescence spectrophotometric titrations were established as detailed prior (λ_ex_ = 448 nm and λ_em_ = 488 nm). For each experiment, three replicate measurements were recorded.

### LOD Measurement

The calculation technique used for the LOD was based on the standard derivation of 10 measurements without the guest molecule (σ) and the slope of the linear calibration curve (K) based on the formula *LOD* = 3σ/*K*.

The standard deviation of 10 measurements without the guest molecule could be determined based on the following relationship: $\sigma =\sqrt{\frac{1}{n-1}\overset{n}{\underset{i=1}{\sum }}{\left(x_{i}-\bar{x}\right)}^{2}}$, where *n* is the number of measurements (*n* = 11).

## Author Contributions

YF, R-HG, YH, and BB undertook the acquisition and analysis of data for the work. ZT and XX drafted the work and revised it critically for important intellectual content.

### Conflict of Interest Statement

The authors declare that the research was conducted in the absence of any commercial or financial relationships that could be construed as a potential conflict of interest.
