# Supplementary material for: Supramolecular Fluorescence Probe Based on Twisted Cucurbit[14]uril for Sensing Fungicide Flusilazole
Source: Front Chem. 2019 Mar 21;7:154. doi: 10.3389/fchem.2019.00154 (PMC6437033; doi:10.3389/fchem.2019.00154)
Supplement: Supplementary file 1 [file Data_Sheet_1.doc]

SUPPORTING INFORMATION

**A supramolecular fluorescence probe based on twisted cucurbit[14]uril for sensing fungicide** **flusilazole**

Ying Fan,^a,b^ Rui-Han Gao,^b^ Ying Huang,^b^ Bing Bian,^c^ Zhu Tao*^b^ and Xin Xiao*^b^

^a^State Key Laboratory Breeding Base of Green Pesticide and Agricultural Bioengineering, Key Laboratory of Green Pesticide and Agricultural Bioengineering, Ministry of Education, Guizhou University, Guiyang 550025, P. R. China

^b^Key Laboratory of Macrocyclic and Supramolecular Chemistry of Guizhou Province, Guizhou University, Guiyang 550025, P. R. China

^c^College of Chemical and Environmental Engineering, Shandong University of Science and Technology, Qingdao 266590, P. R. China

E-mail: gyhxxiaoxin@163.com (X. Xiao) and gzutao@263.net (Z. Tao)

**TABLE OF CONTENTS**

**Figure S1** Fluorescence titration spectra (λ_ex_ = 448 nm) of ThT@*t*Q[14] (1:1, 1 *μ*M) in the presence of different stoichiometries of triazole pesticides / *μ*M: 0, 0.25, 0.5, 0.75, 1.0, 1.25, 1.5, 1.75 and 2.0. (a) azaconazole, (b) triadimefon, (c) tebuconazole, (d) tricyclazole, (e) flutriafol, (f) penconazole and (g) triadimenol isomer A, respectively.

**Figure S2** (left) Fluorescence titration spectra (λ_ex_ = 448 nm) of ThT@*t*Q[14] (1:5, 1 *μ*M) in the presence of different stoichiometries of flusilazole / *μ*M: 0, 0.25, 0.5, 0.75, 1.0, 1.25, 1.5, 1.75, 2.0, 2.5, 3.0, 3.5 and 4.0; (right) Profile of fluorescence intensity of ThT@*t*Q[14] *vs* C_flusilazole_/C_ThT_*_@t_*_Q[14]_.

**Figure S3** Fluorescence titration spectra (λ_ex_ = 448 nm) of ThT@*t*Q[14] (1:5, 1 *μ*M) in the presence of different stoichiometries of triazole pesticides / *μ*M: 0, 0.25, 0.5, 0.75, 1.0, 1.25, 1.5, 1.75 and 2.0. (a) azaconazole, (b) triadimefon, (c) tebuconazole, (d) tricyclazole, (e) flutriafol, (f) penconazole and (g) triadimenol isomer A, respectively.

**Figure S4** Nonlinear fitted fluorescence data for *t*Q[14]−ThT (1:1, 1 *μ*M) with flusilazole.

**Figure S5** Standard calibration curves of the complexes of flusilazole with ThT*@t*Q[14] (1:1).

**Figure S6** Standard calibration curves of the complexes of flusilazole with ThT*@t*Q[14] (1:5).

**Figure S7** Fluorescence titration spectra (λ_ex_ = 448 nm) of *t*Q[14]−ThT−flusilazole (1:1:1, 1 *μ*M) in the presence of different stoichiometries (a) Na^+^, (b) K^+^, (c) Zn^2+^, (d) Mg^2+^, (e) Cu^2+^, (f) Cl^−^, (g) Br^−^, (h) NO_3_^−^, (i) HSO_4_^−^ and (j) H_2_PO_4_^−^.

**Figure S8** (left) Fluorescence titration spectra (λ_ex_ = 448 nm) of *t*Q[14]−ThT−flusilazole (1:1:1, 1 *μ*M) in the presence of different stoichiometries of Ca^2+^ / *μ*M: 0, 10, 20, 40, 60, 80, 100, 120, 140, 160, 180 and 200; (right) Profile of fluorescence intensity of *t*Q[14]-ThT-flusilazole *vs* C_Ca_^2+^/C*_t_*_Q[14]-ThT-flusilazole_.

**Figure S9** Titration ^1^H NMR spectra (400 MHz, D_2_O) of (a) ThT, (b) *t*Q[14]–ThT (1:1), (c) *t*Q[14]–ThT–azaconazole (1:1:1), (d-i) *t*Q[14]–ThT–azaconazole (1:1:1) in the presence of Ca^2+^.

**Figure S10** ^1^H NMR spectra (400 MHz, D_2_O) of (a) ThT, (b) *t*Q[14]–ThT (1:1), (c) *t*Q[14]–ThT–azaconazole (1:1:0.5), (d) *t*Q[14]–ThT–azaconazole (1:1:1), (e) *t*Q[14]–ThT–azaconazole (1:1:2), (f) *t*Q[14]–azaconazole and (g) azaconazole.

**Figure S11** ^1^H NMR spectra (400 MHz, D_2_O) of (a) ThT, (b) *t*Q[14]–ThT (1:1), (c) *t*Q[14]–ThT–triadimefon (1:1:1), (d) *t*Q[14]–ThT–triadimefon (1:1:2), (e) *t*Q[14]–triadimefon and (f) triadimefon.

**Figure S12** ^1^H NMR spectra (400 MHz, D_2_O) of (a) ThT, (b) *t*Q[14]–ThT (1:1), (c) *t*Q[14]–ThT–tebuconazole (1:1:1), (d) *t*Q[14]–ThT–tebuconazole (1:1:2), (e) *t*Q[14]–tebuconazole and (f) tebuconazole.

**Figure S13** ^1^H NMR spectra (400 MHz, D_2_O) of (a) ThT, (b) *t*Q[14]–ThT (1:1), (c) *t*Q[14]–ThT–tricyclazole (1:1:1), (d) *t*Q[14]–ThT–tricyclazole (1:1:2), (e) *t*Q[14]–tricyclazole and (f) tricyclazole.

**Figure S14** ^1^H NMR spectra (400 MHz, D_2_O) of (a) ThT, (b) *t*Q[14]–ThT (1:1), (c) *t*Q[14]–ThT–flutriafol (1:1:1), (d) *t*Q[14]–ThT–flutriafol (1:1:2), (e) *t*Q[14]–flutriafol and (f) flutriafol.

**Figure S15** ^1^H NMR spectra (400 MHz, D_2_O) of (a) ThT, (b) *t*Q[14]–ThT (1:1), (c) *t*Q[14]–ThT–penconazole (1:1:1), (d) *t*Q[14]–ThT–penconazole (1:1:1), (e) *t*Q[14]–penconazole and (f) penconazole.

**Figure S16** ^1^H NMR spectra (400 MHz, D_2_O) of (a) ThT, (b) *t*Q[14]–ThT (1:1), (c) *t*Q[14]–ThT–triadimenol isomer A (1:1:1), (d) *t*Q[14]–ThT–triadimenol isomer A (1:1:2), (e) *t*Q[14]–triadimenol isomer A and (f) triadimenol isomer A.

**Figure S17** The DOSY spectra of *t*Q[14], ThT, flusilazole and *t*Q[14]-ThT-flusilazole (1:1:1) ternary interaction species in D_2_O at 298 K.

**Figure S18** Isothermal titration calorimetry profiles of *t*Q[14] in the presence of the guest ThT in aqueous solution at 298.15 K.

**Figure S19** Isothermal titration calorimetry profiles of *t*Q[14] in the presence of the guest flusilazole in aqueous solution at 298.15 K.

**Figure S20** Isothermal titration calorimetry profiles of *t*Q[14] in the presence of the guest azaconazole in aqueous solution at 298.15 K.

**Figure S21** Isothermal titration calorimetry profiles of *t*Q[14] in the presence of the guest triadimefon in aqueous solution at 298.15 K.

**Figure S22** Isothermal titration calorimetry profiles of *t*Q[14] in the presence of the guest tebuconazole in aqueous solution at 298.15 K.

**Figure S23** Isothermal titration calorimetry profiles of *t*Q[14] in the presence of the guest tricyclazole in aqueous solution at 298.15 K.

**Figure S24** Isothermal titration calorimetry profiles of *t*Q[14] in the presence of the guest flutriafol in aqueous solution at 298.15 K.

**Figure S25** Isothermal titration calorimetry profiles of *t*Q[14] in the presence of the guest penconazole in aqueous solution at 298.15 K.

**Figure S26** Isothermal titration calorimetry profiles of *t*Q[14] in the presence of the guest triadimenol isomer A in aqueous solution at 298.15 K.

**Figure S27** Fluorescence spectra (λ_ex_=448 nm) for ThT@HMeQ[6] (1:1, 1 *μ*M) and ThT@Q[7] (1:1, 1 *μ*M).


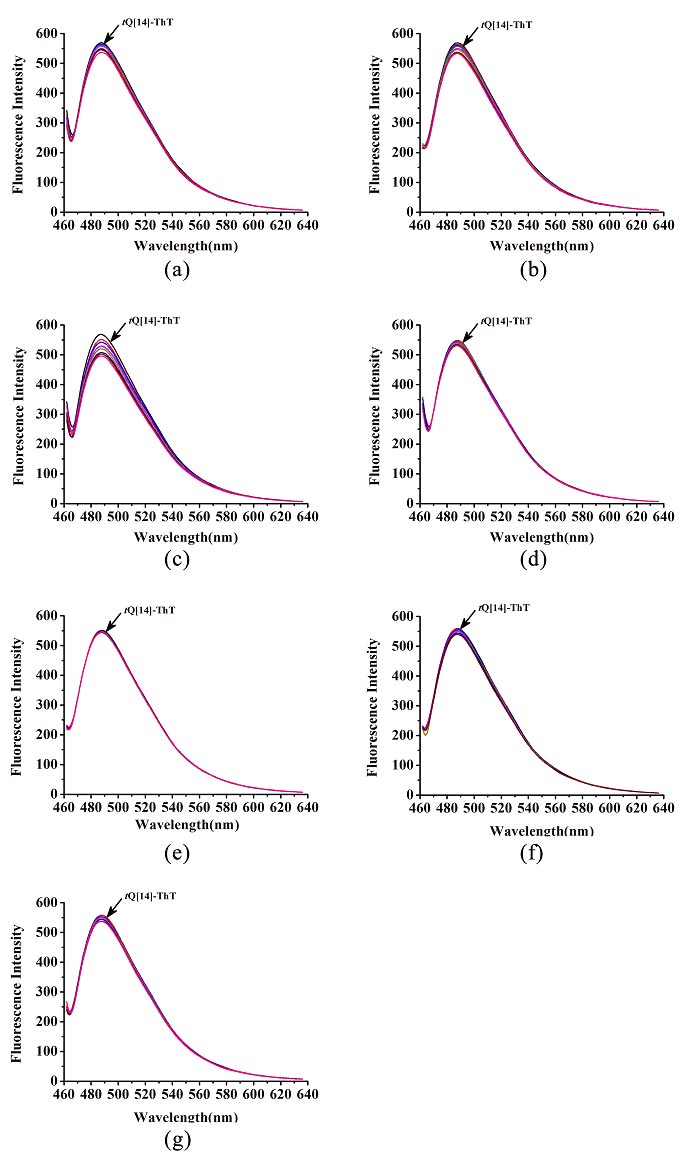


**Figure S1** Fluorescence titration spectra (λ_ex_ = 448 nm) of ThT@*t*Q[14] (1:1, 1 *μ*M) in the presence of different stoichiometries of triazole pesticides / *μ*M: 0, 0.25, 0.5, 0.75, 1.0, 1.25, 1.5, 1.75 and 2.0. (a) azaconazole, (b) triadimefon, (c) tebuconazole, (d) tricyclazole, (e) flutriafol, (f) penconazole and (g) triadimenol isomer A, respectively.

**
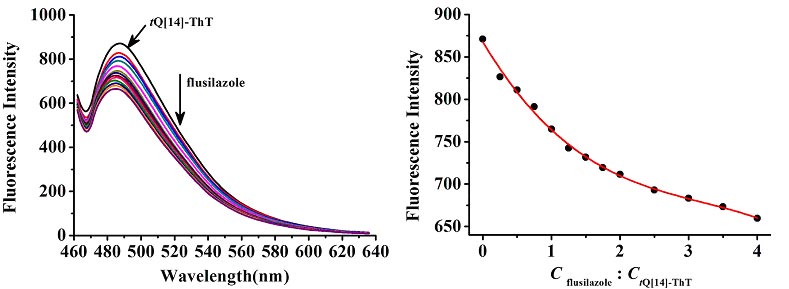
**

**Figure S2** (left) Fluorescence titration spectra (λ_ex_ = 448 nm) of ThT@*t*Q[14] (1:5, 1 *μ*M) in the presence of different stoichiometries of flusilazole / *μ*M: 0, 0.25, 0.5, 0.75, 1.0, 1.25, 1.5, 1.75, 2.0, 2.5, 3.0, 3.5 and 4.0; (right) Profile of fluorescence intensity of ThT@*t*Q[14] *vs* C_flusilazole_/C_ThT_*_@t_*_Q[14]_.

**
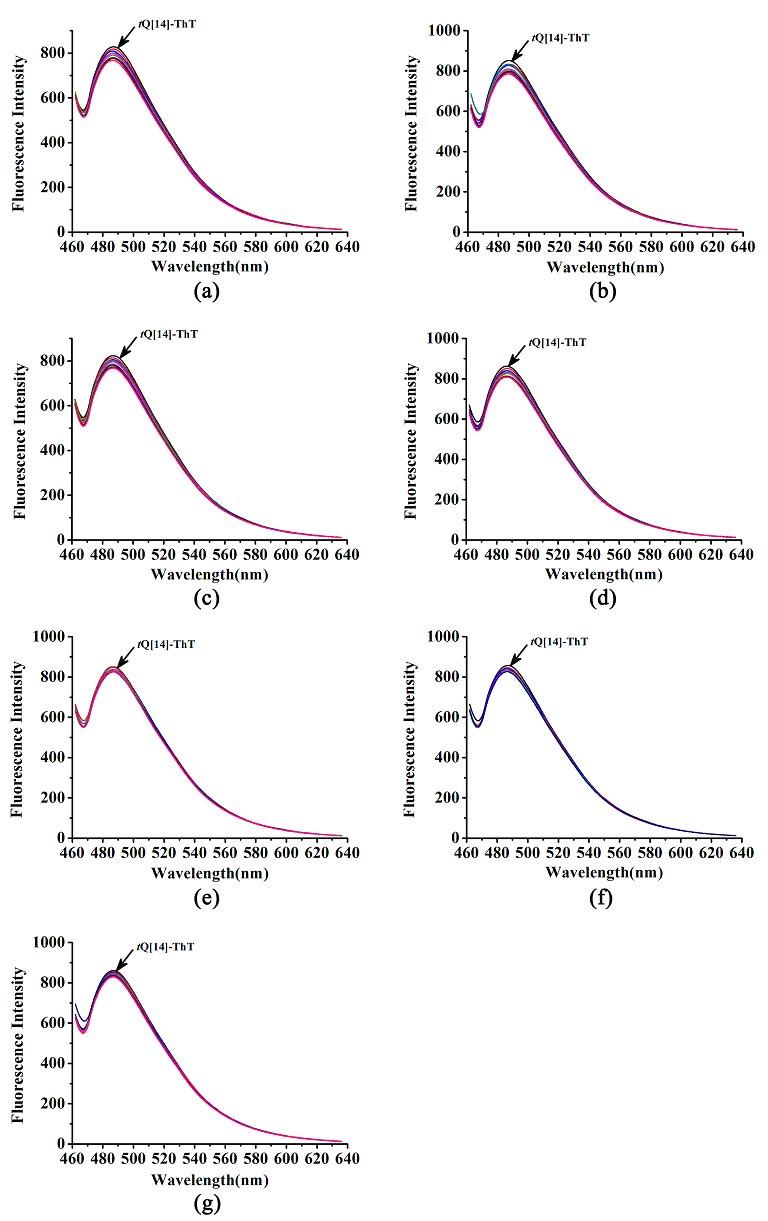
**

**Figure S3** Fluorescence titration spectra (λ_ex_ = 448 nm) of ThT@*t*Q[14] (1:5, 1 *μ*M) in the presence of different stoichiometries of triazole pesticides / *μ*M: 0, 0.25, 0.5, 0.75, 1.0, 1.25, 1.5, 1.75 and 2.0. (a) azaconazole, (b) triadimefon, (c) tebuconazole, (d) tricyclazole, (e) flutriafol, (f) penconazole and (g) triadimenol isomer A, respectively.

**
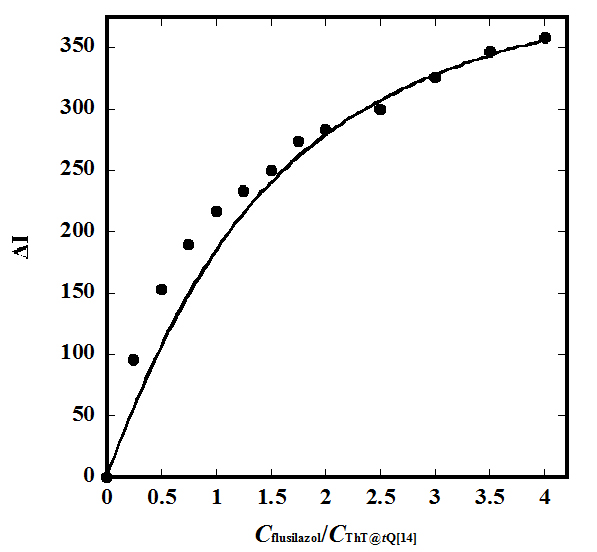
**

**Figure S4** Nonlinear fitted fluorescence data for *t*Q[14]−ThT (1:1, 1 *μ*M) with flusilazole.

**Figure S5** Standard calibration curves of the complexes of flusilazole with ThT*@t*Q[14] (1:1).

**Figure S6** Standard calibration curves of the complexes of flusilazole with ThT*@t*Q[14] (1:5).

**
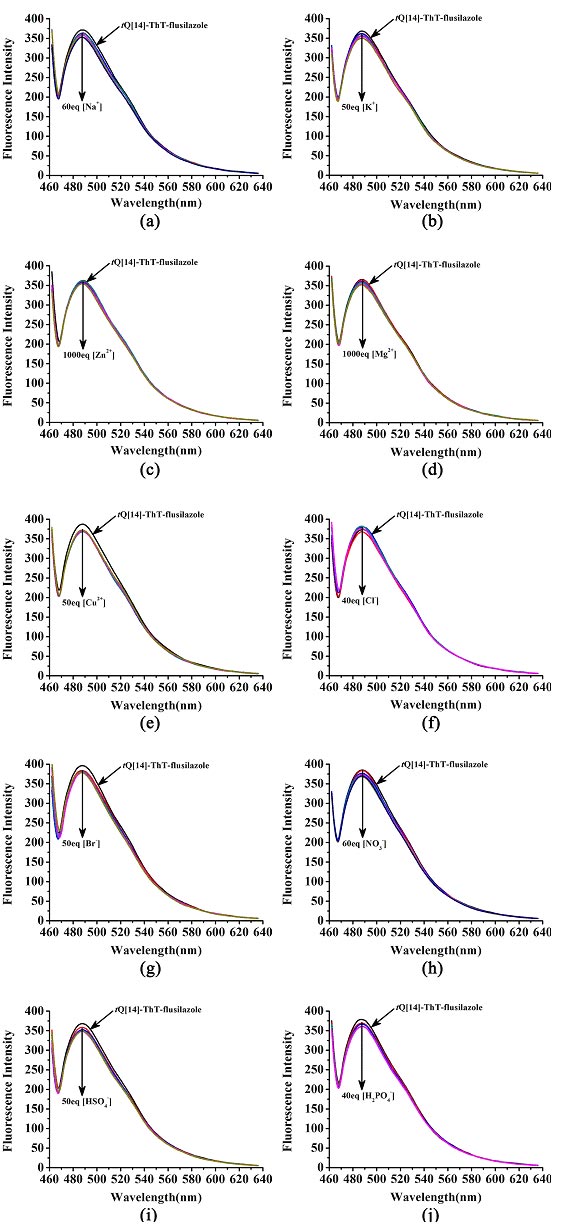
**

**Figure S7** Fluorescence titration spectra (λ_ex_ = 448 nm) of *t*Q[14]−ThT−flusilazole (1:1:1, 1 *μ*M) in the presence of different stoichiometries (a) Na^+^, (b) K^+^, (c) Zn^2+^, (d) Mg^2+^, (e) Cu^2+^, (f) Cl^−^, (g) Br^−^, (h) NO_3_^−^, (i) HSO_4_^−^ and (j) H_2_PO_4_^−^.

**
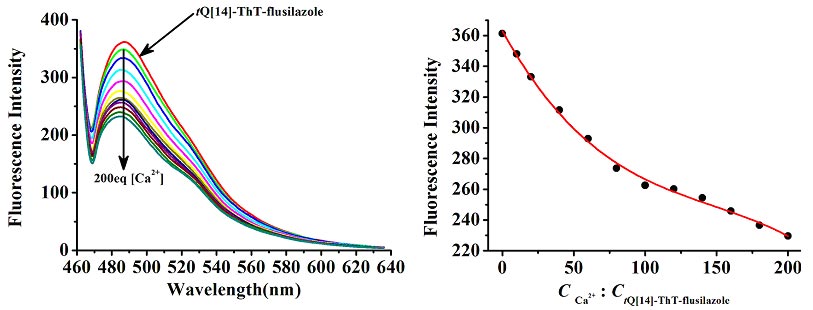
**

**Figure S8** (left) Fluorescence titration spectra (λ_ex_ = 448 nm) of *t*Q[14]−ThT−flusilazole (1:1:1, 1 *μ*M) in the presence of different stoichiometries of Ca^2+^ / *μ*M: 0, 10, 20, 40, 60, 80, 100, 120, 140, 160, 180 and 200; (right) Profile of fluorescence intensity of *t*Q[14]-ThT-flusilazole *vs* C_Ca_^2+^/C*_t_*_Q[14]-ThT-flusilazole_.

**
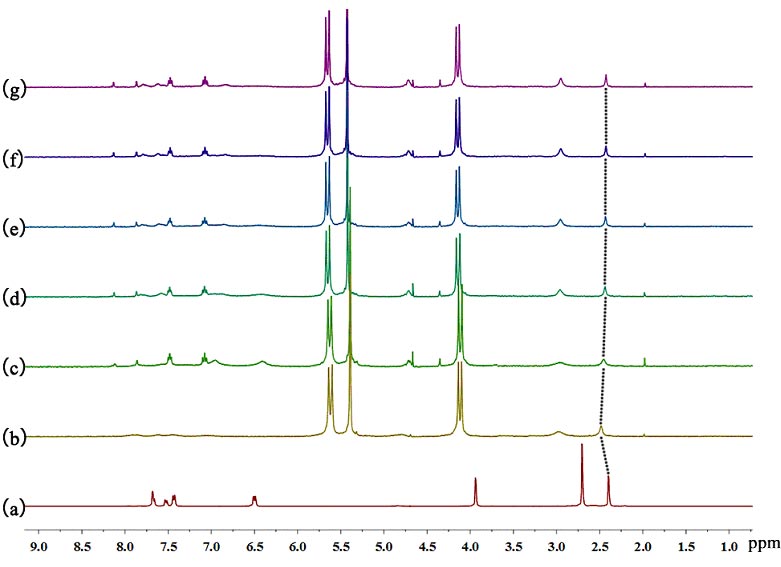
**

**Figure S9** Titration ^1^H NMR spectra (400 MHz, D_2_O) of (a) ThT, (b) *t*Q[14]–ThT (1:1), (c) *t*Q[14]–ThT–azaconazole (1:1:1), (d-g) *t*Q[14]–ThT–azaconazole (1:1:1) in the presence of Ca^2+^.

**
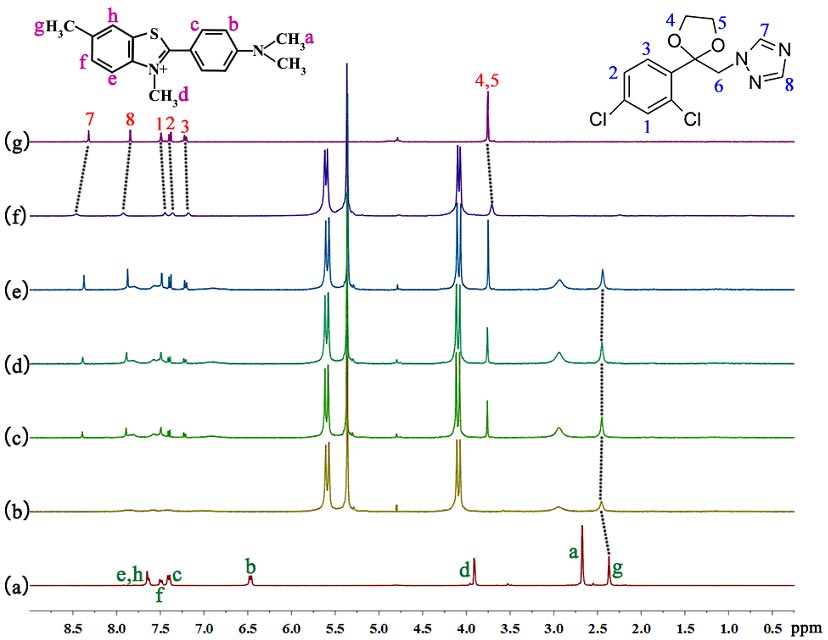
**

**Figure S10** ^1^H NMR spectra (400 MHz, D_2_O) of (a) ThT, (b) *t*Q[14]–ThT (1:1), (c) *t*Q[14]–ThT–azaconazole (1:1:0.5), (d) *t*Q[14]–ThT–azaconazole (1:1:1), (e) *t*Q[14]–ThT–azaconazole (1:1:2), (f) *t*Q[14]–azaconazole and (g) azaconazole.

**
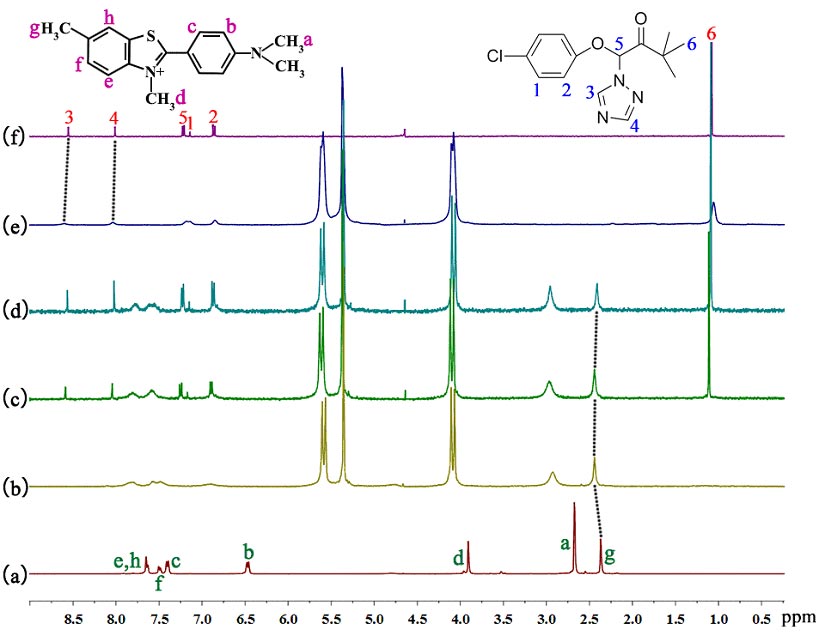
**

**Figure S11** ^1^H NMR spectra (400 MHz, D_2_O) of (a) ThT, (b) *t*Q[14]–ThT (1:1), (c) *t*Q[14]–ThT–triadimefon (1:1:1), (d) *t*Q[14]–ThT–triadimefon (1:1:2), (e) *t*Q[14]–triadimefon and (f) triadimefon.

**
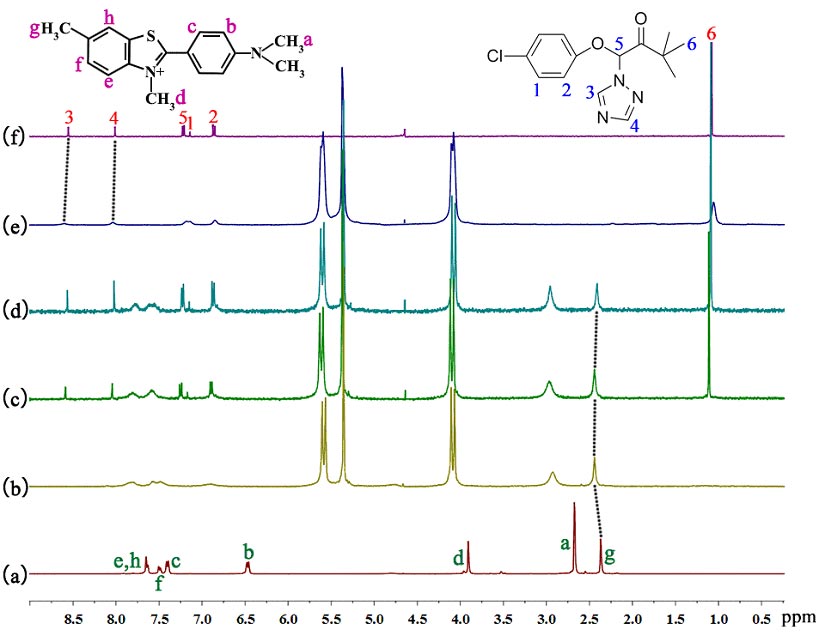
**

**Figure S12** ^1^H NMR spectra (400 MHz, D_2_O) of (a) ThT, (b) *t*Q[14]–ThT (1:1), (c) *t*Q[14]–ThT–tebuconazole (1:1:1), (d) *t*Q[14]–ThT–tebuconazole (1:1:2), (e) *t*Q[14]–tebuconazole and (f) tebuconazole.


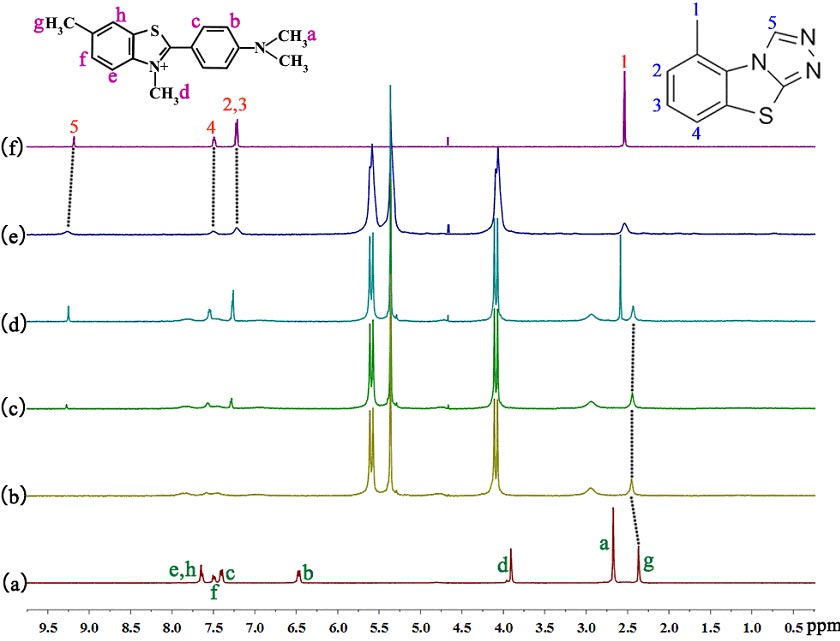


**Figure S13** ^1^H NMR spectra (400 MHz, D_2_O) of (a) ThT, (b) *t*Q[14]–ThT (1:1), (c) *t*Q[14]–ThT–tricyclazole (1:1:1), (d) *t*Q[14]–ThT–tricyclazole (1:1:2), (e) *t*Q[14]–tricyclazole and (f) tricyclazole.

**
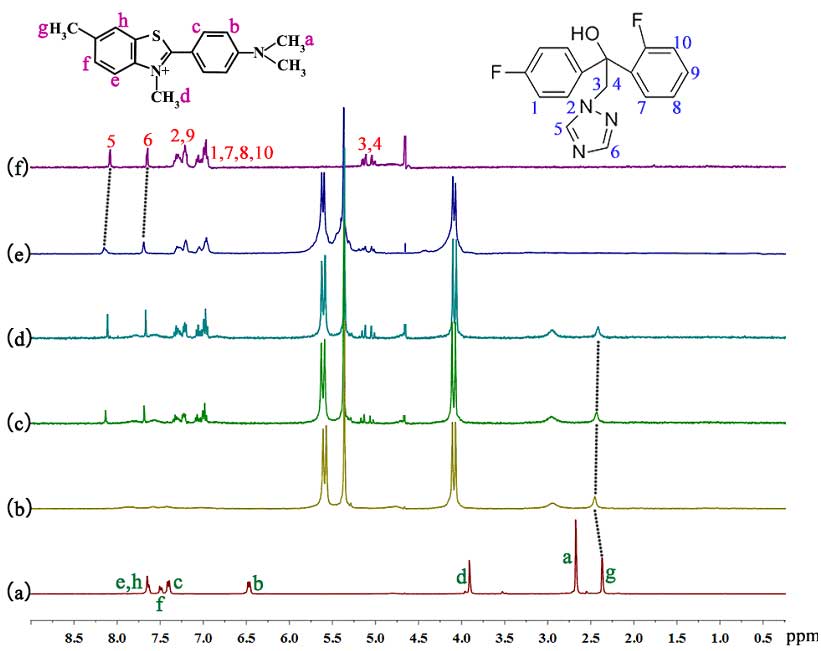
**

**Figure S14** ^1^H NMR spectra (400 MHz, D_2_O) of (a) ThT, (b) *t*Q[14]–ThT (1:1), (c) *t*Q[14]–ThT–flutriafol (1:1:1), (d) *t*Q[14]–ThT–flutriafol (1:1:2), (e) *t*Q[14]–flutriafol and (f) flutriafol.

**
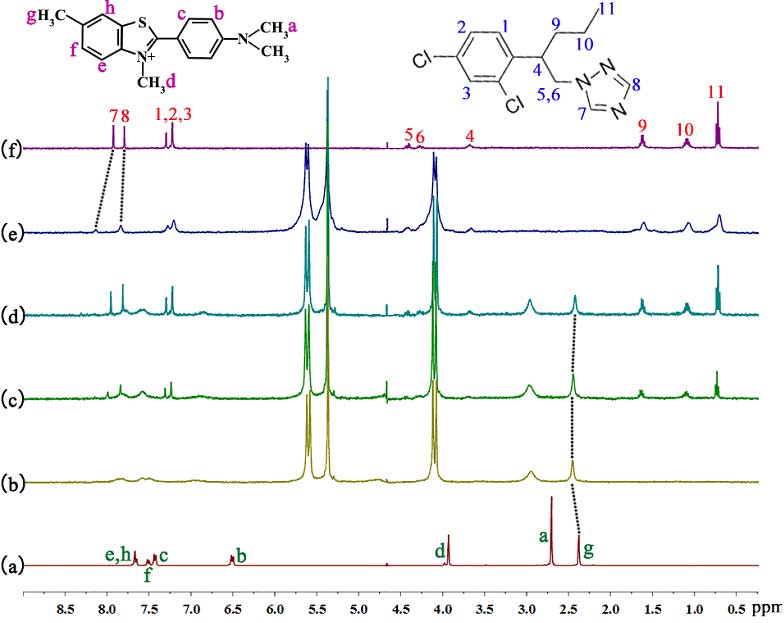
**

**Figure S15** ^1^H NMR spectra (400 MHz, D_2_O) of (a) ThT, (b) *t*Q[14]–ThT (1:1), (c) *t*Q[14]–ThT–penconazole (1:1:1), (d) *t*Q[14]–ThT–penconazole (1:1:1), (e) *t*Q[14]–penconazole and (f) penconazole.

**
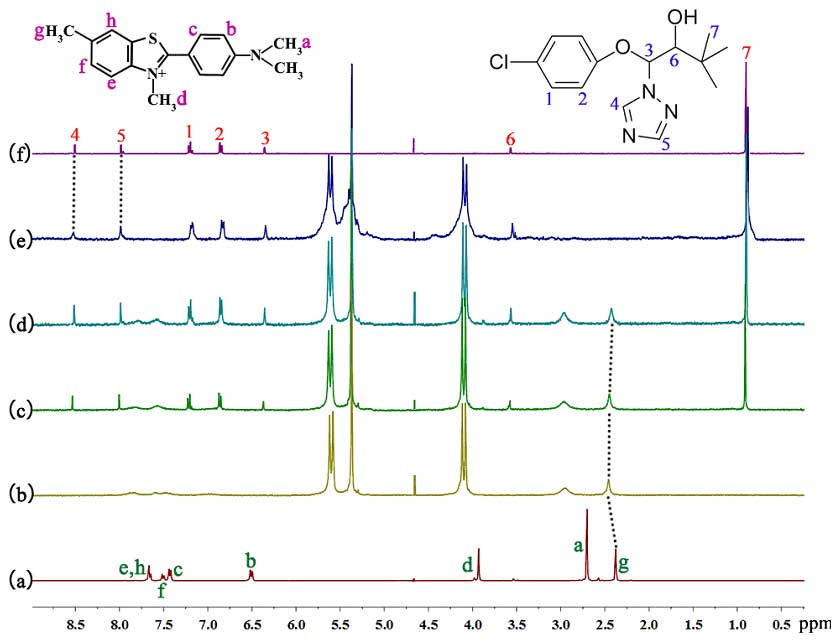
**

**Figure S16** ^1^H NMR spectra (400 MHz, D_2_O) of (a) ThT, (b) *t*Q[14]–ThT (1:1), (c) *t*Q[14]–ThT–triadimenol isomer A (1:1:1), (d) *t*Q[14]–ThT–triadimenol isomer A (1:1:2), (e) *t*Q[14]–triadimenol isomer A and (f) triadimenol isomer A.

*t*Q[14]


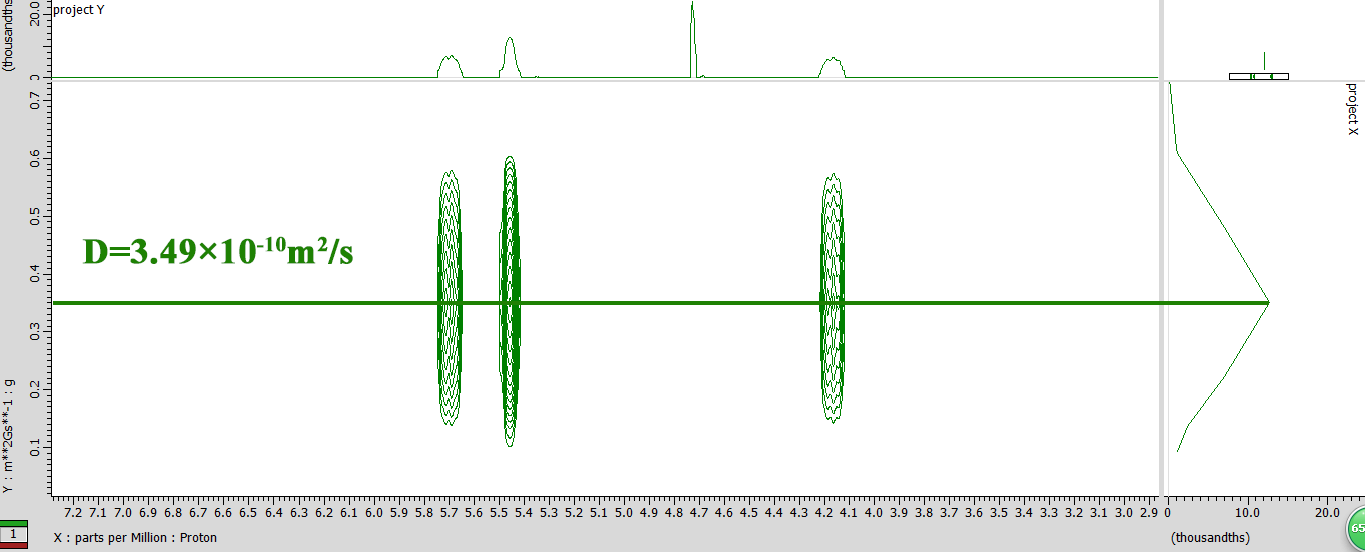


ThT


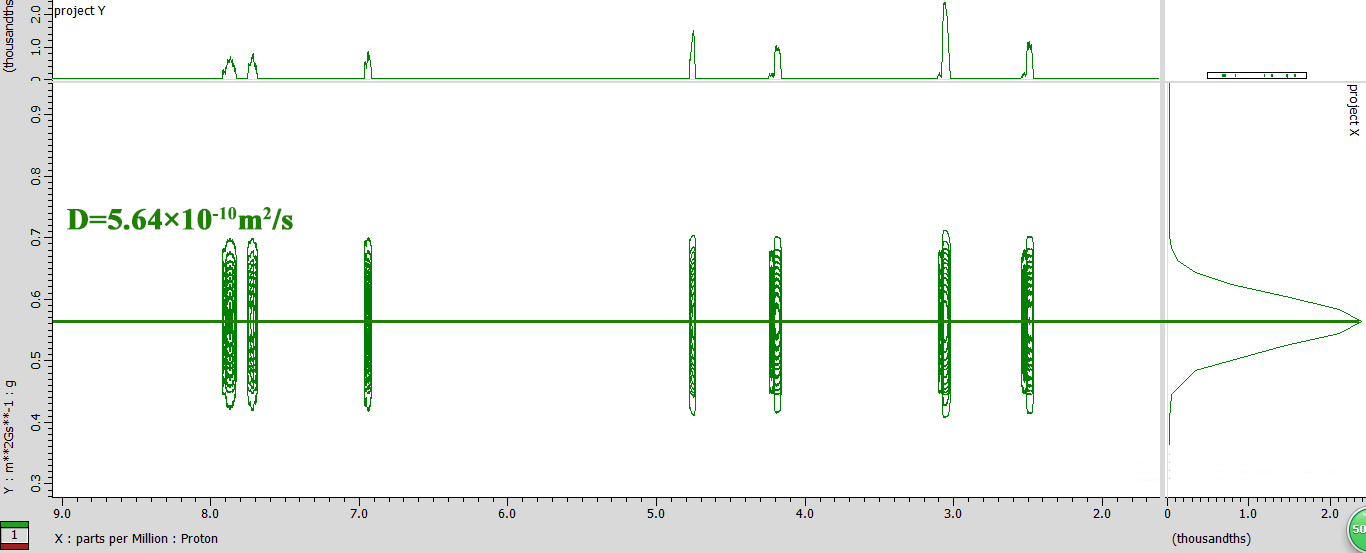


flusilazole


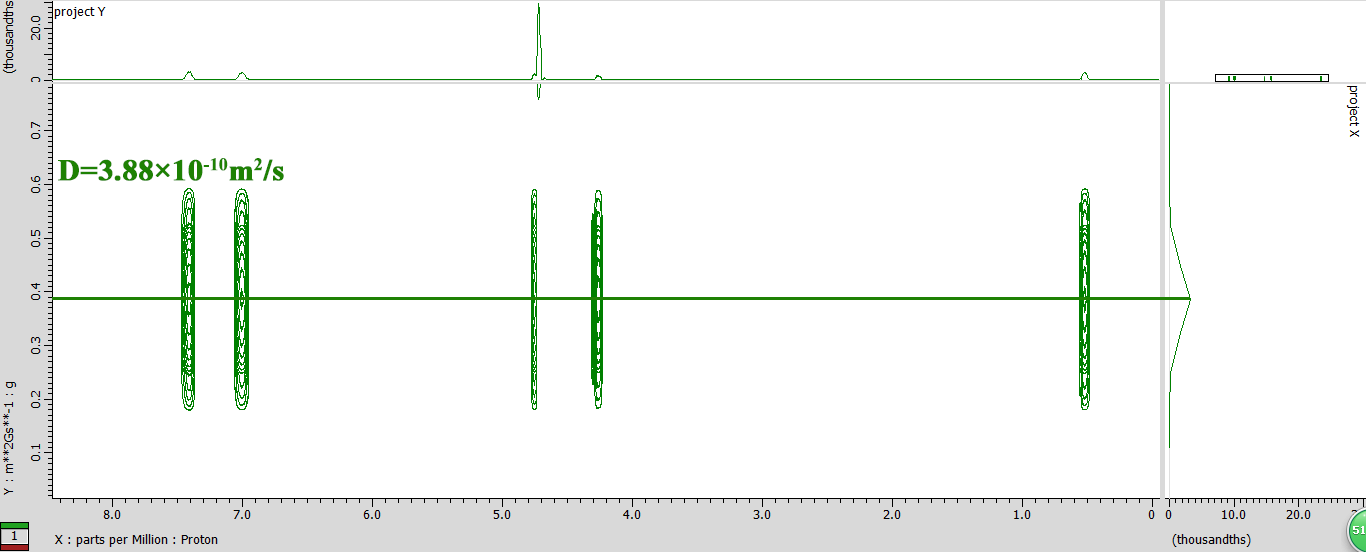


*t*Q[14]-ThT- flusilazole（1:1:1）


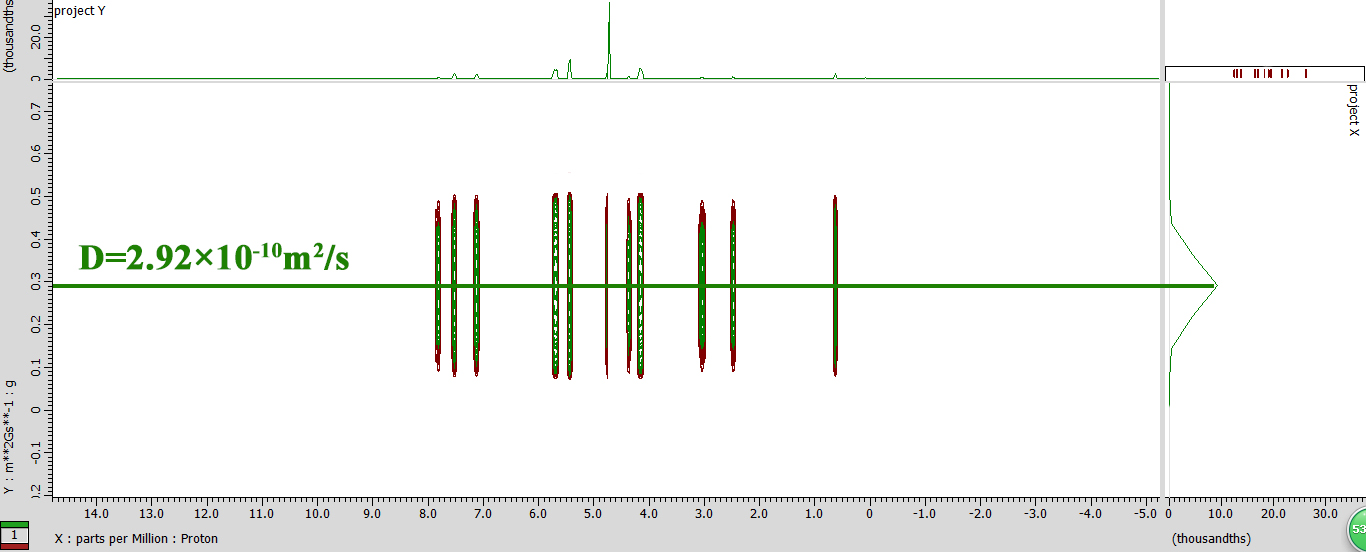


**Figure S17** The DOSY spectra of *t*Q[14], ThT, flusilazole and *t*Q[14]-ThT-flusilazole (1:1:1) ternary interaction species in D_2_O at 298 K.


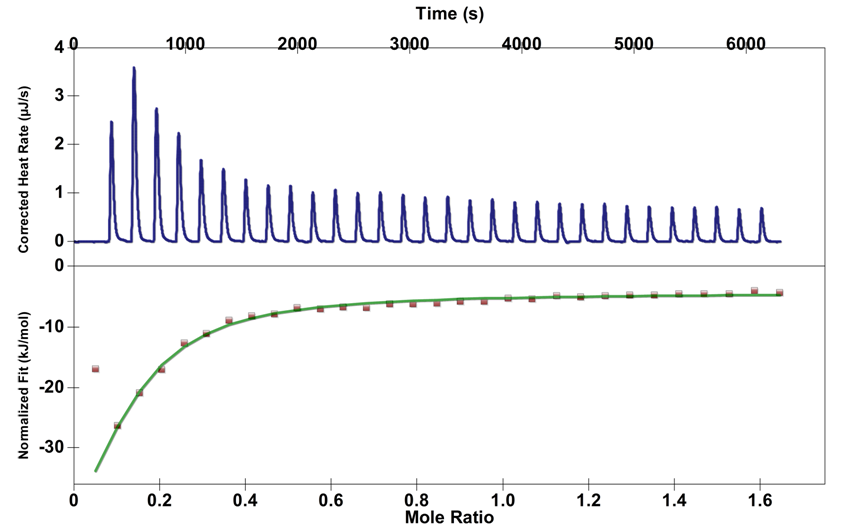


**Figure S18** Isothermal titration calorimetry profiles of *t*Q[14] in the presence of the guest ThT in aqueous solution at 298.15 K.


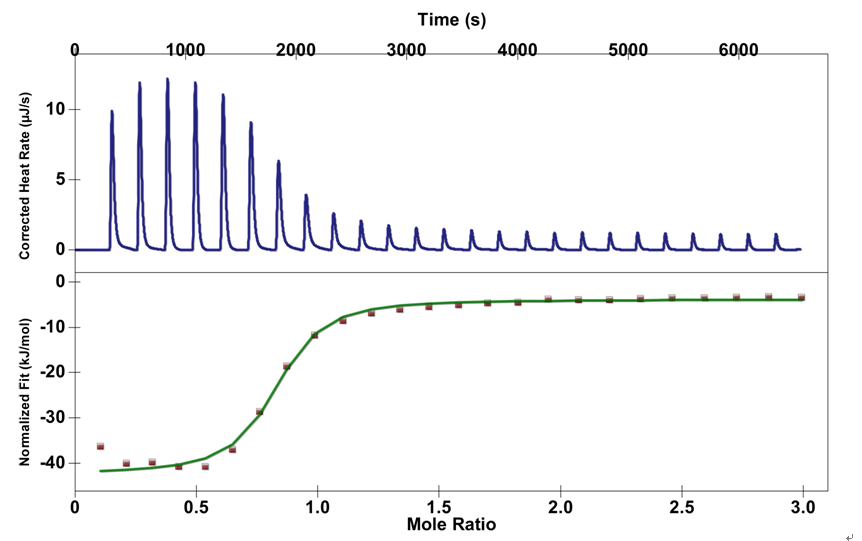


**Figure S19** Isothermal titration calorimetry profiles of *t*Q[14] in the presence of the guest flusilazole in aqueous solution at 298.15 K.


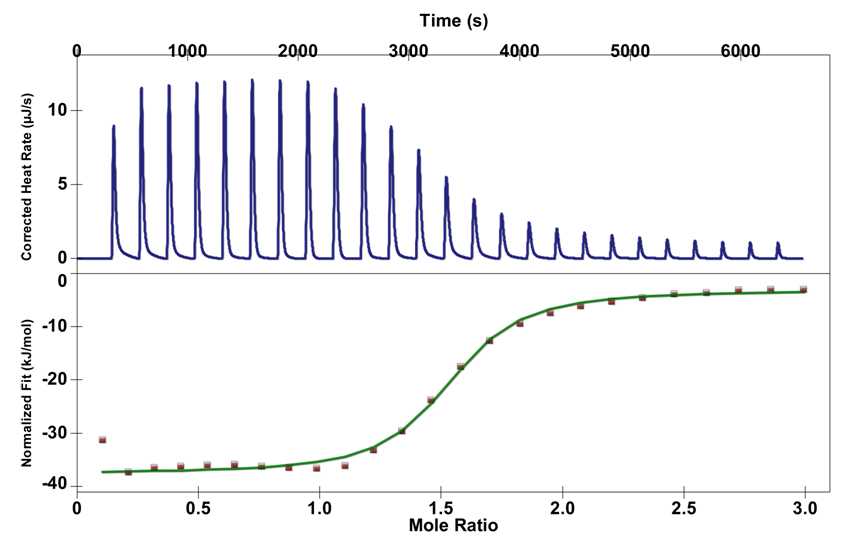


**Figure S20** Isothermal titration calorimetry profiles of *t*Q[14] in the presence of the guest azaconazole in aqueous solution at 298.15 K.

**
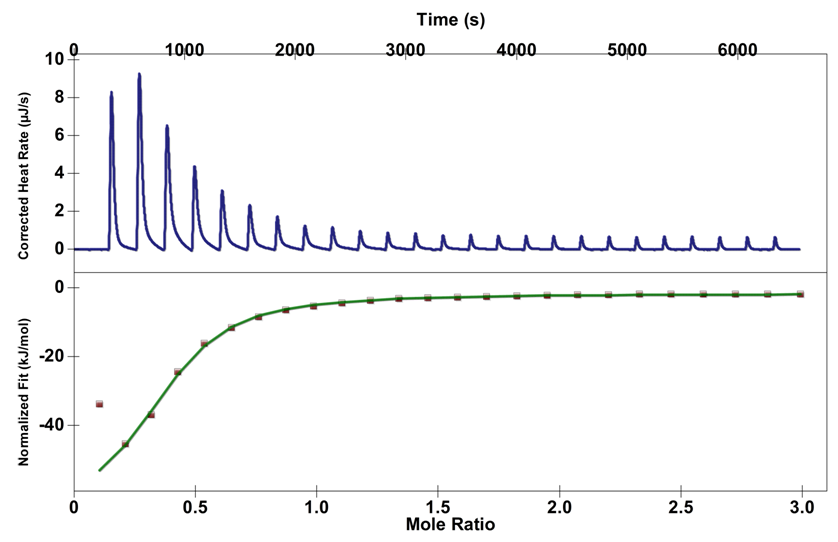
Figure S21** Isothermal titration calorimetry profiles of *t*Q[14] in the presence of the guest triadimefon in aqueous solution at 298.15 K.

**
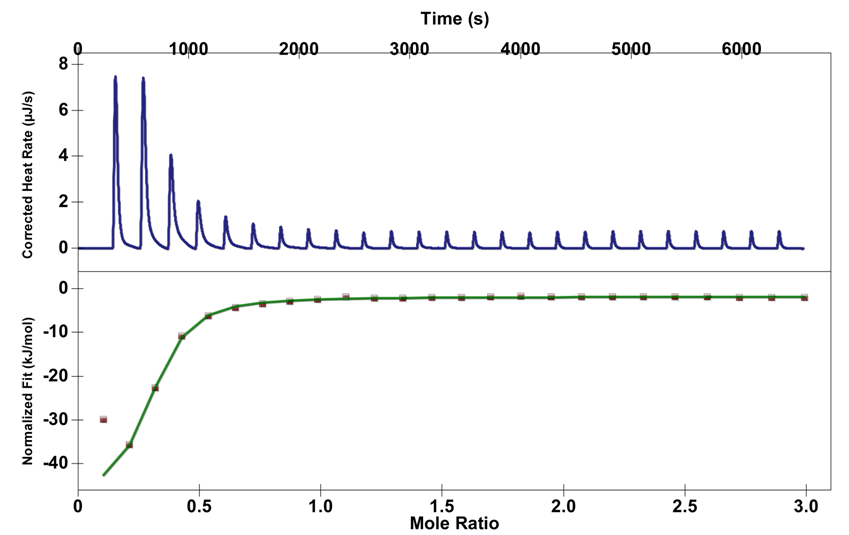
**

**Figure S22** Isothermal titration calorimetry profiles of *t*Q[14] in the presence of the guest tebuconazole in aqueous solution at 298.15 K.


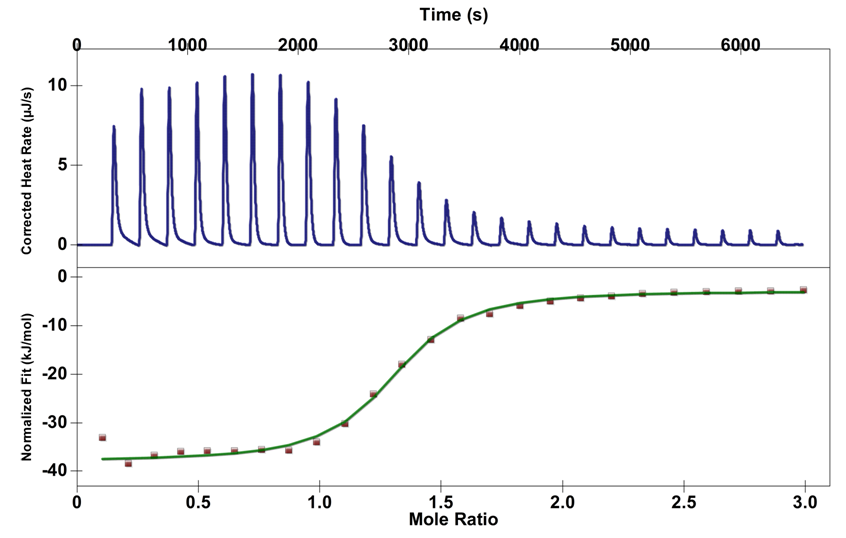


**Figure S23** Isothermal titration calorimetry profiles of *t*Q[14] in the presence of the guest tricyclazole in aqueous solution at 298.15 K.


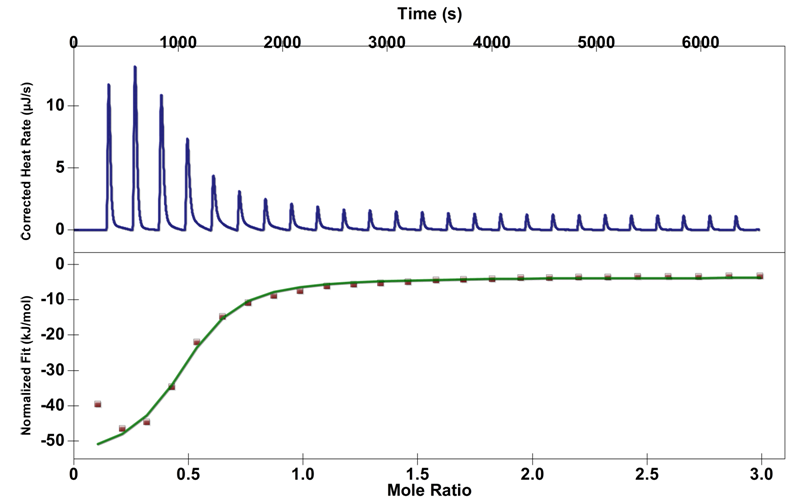


**Figure S24** Isothermal titration calorimetry profiles of *t*Q[14] in the presence of the guest flutriafol in aqueous solution at 298.15 K.


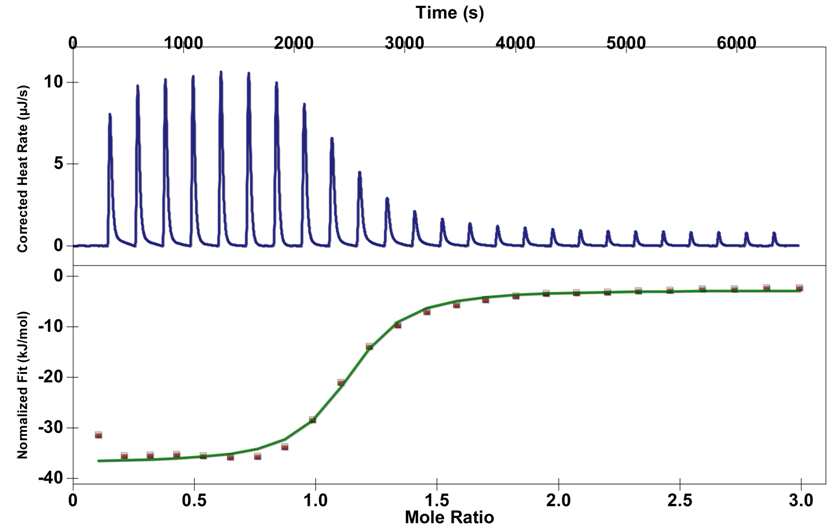


**Figure S25** Isothermal titration calorimetry profiles of *t*Q[14] in the presence of the guest penconazole in aqueous solution at 298.15 K.


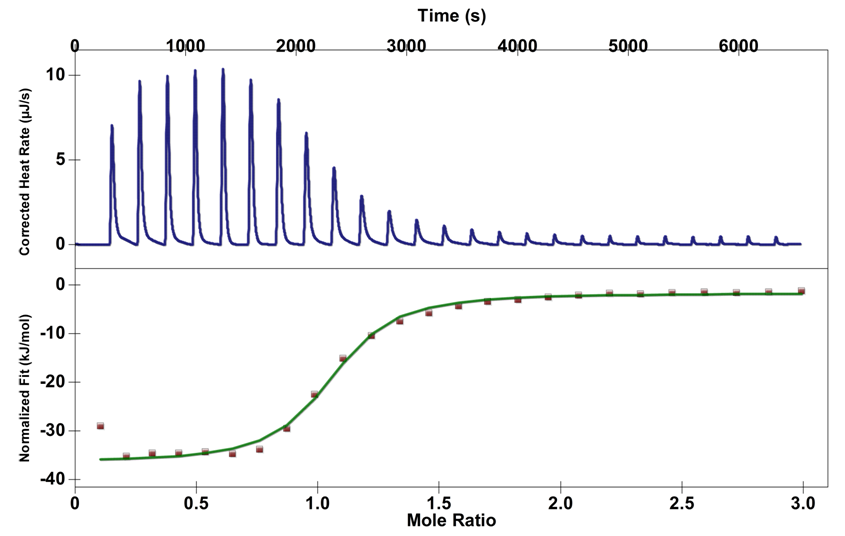


**Figure S26** Isothermal titration calorimetry profiles of *t*Q[14] in the presence of the guest triadimenol isomer A in aqueous solution at 298.15 K.


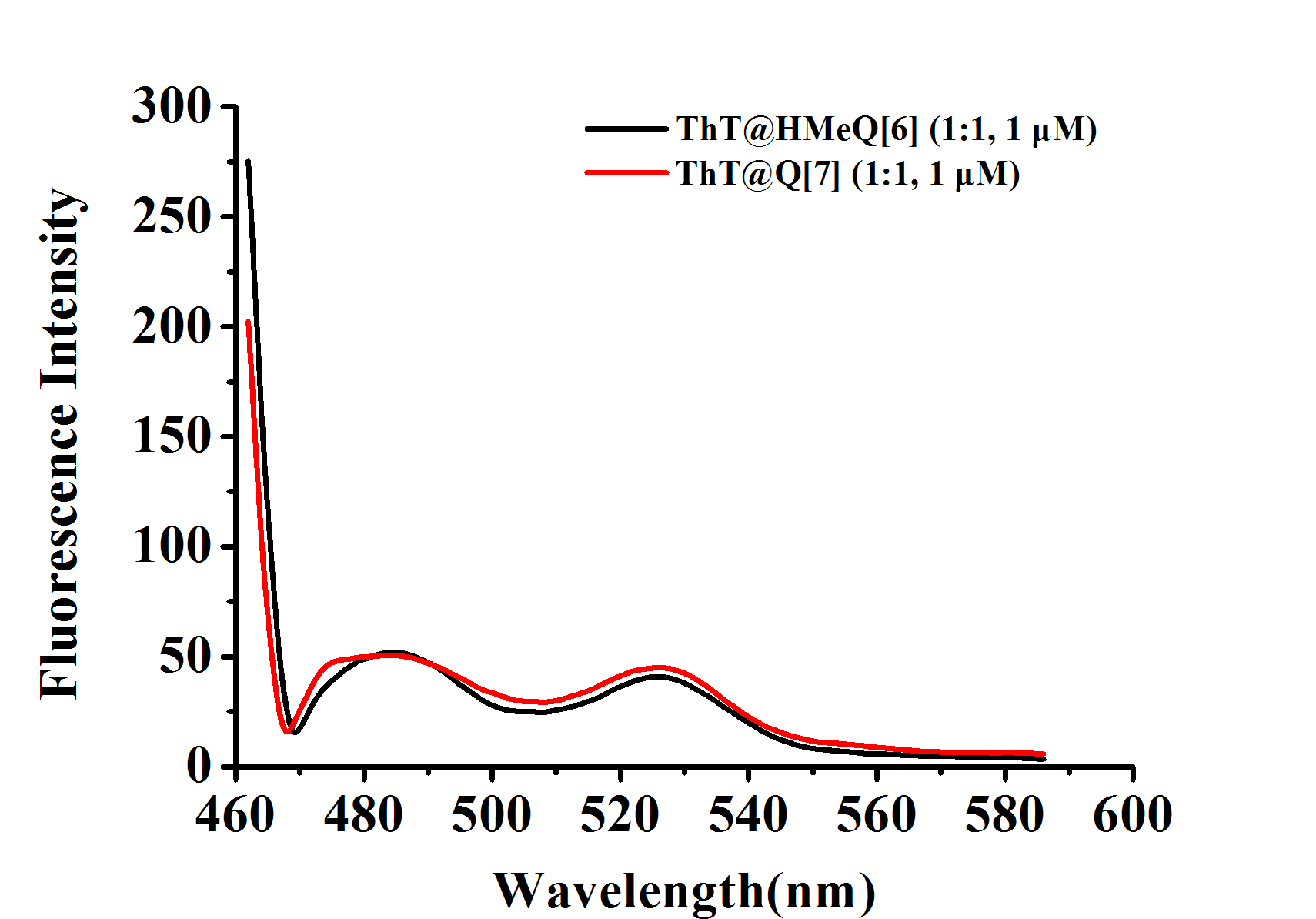


**Figure S27** Fluorescence spectra (λ_ex_=448 nm) for ThT@HMeQ[6] (1:1, 1 *μ*M) and ThT@Q[7] (1:1, 1 *μ*M).
